# The contribution of medical physics to nuclear medicine: a physician's perspective

**DOI:** 10.1186/2197-7364-1-3

**Published:** 2014-05-01

**Authors:** Peter J Ell

**Affiliations:** Institute of Nuclear Medicine (T5), University College London NHS Trust Hospitals, 235 Euston Road, London, NW1 2BU UK

**Keywords:** Nuclear medicine, Physics, History

## Abstract

This paper is the second in a series of invited perspectives by four pioneers of nuclear medicine imaging and physics. A medical physicist and a nuclear medicine clinical specialist each take a backward look and a forward look at the contributions of physics to nuclear medicine. Here is a backward look from a nuclear medicine physician's perspective.

“*He who does not doubt, does not investigate, does not perceive; and he who does not perceive, remains in blindness and error”*

Al-Ghazali (1058–1111 a.c.), theologian, jurist, philosopher, cosmologist, psychologist and mystic

The introduction of radioactive tracers to clinical medicine can be traced to the late 40 s [1–3]. From its inception, physicians and physicists made use of purposely developed detection instruments and radionuclides in order to (a) further the understanding of the underlying mechanisms of disease in man and (b) to investigate the earliest manifestations of pathologies. To diagnose early on and to treat if possible were mutual aims of both physicians and physicists. To underline, the contribution of scientists and physicists to the development of Nuclear Medicine has been not just major but disruptive and of fundamental importance. The very first applications preceded the previously mentioned by half a century (Table 1).

**Table 1:** **The pioneers of nuclear medicine**

1895	X-rays	Wilhelm C. Roentgen	German physicist	1845 to 1923
1896	Radioactivity	Antoine H. Becquerel	French physicist	1852 to 1908
1898	Polonium, radium,thorium	Marie Sklodowska Curie	French physicist	1867 to 1934
1923	Tracer principle	Georg V. Hevesy	Hungarian chemist	1885 to 1966
1927	Circulation times	Hermann L. Blumgart	German doctor	1895 to 1977
1928	Counter	Johannes W. Geiger	German physicist	1882 to 1945
		Walther Mueller	German physicist	1905 to 1979
1932	Cyclotron	Ernest O. Lawrence	American physicist	1901 to 1958

## November 12, 1936

Visiting patients at his thyroid clinic at Mass General Hospital, the physician JH Means, M.D., poses a most relevant question. In his mind was already the understanding of the role and importance of iodine metabolism of the thyroid and the possibility to measure it non-invasively *in vivo*. RE Evans, Ph.D., rose to the challenge with a seminar pronouncement:

JH Means M.D.: ‘Is there a radioisiotope of iodine?’

Robley Evans, Ph.D.: Mass. Inst. Techn.: ‘We can make some’.

**Figure 1:**
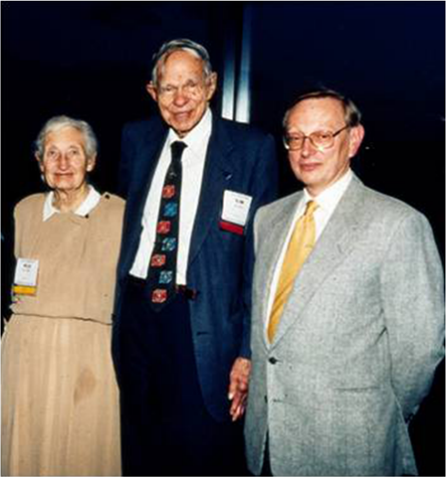
**Glenn Seaborg (middle) and wife (left), together with the author (right) at a SNM meeting in the late 1990s.** Seaborg won the 1951 Nobel Prize for Chemistry, discovered over 100 isotopes, advised 10 Presidents of the USA and was Chairman of the USA Atomic Energy Commission for 10 years.

This seminal encounter possibly marks the development of what was to become known as Nuclear Medicine (Table 2, Figure 1). It is the perfect example of the interdisciplinary thinking which was to permeate and characterise the development of this speciality. It also represents the ideal setup, where an identified problem, namely, the investigation of thyroid physiopathology, led to the development of a new investigative tool (the radionuclide). It would take many years indeed before another ‘magic bullet’ was to be identified and widely applied. If specificity is intended by such a magic bullet, receptor ligands such as those targeting the dopamine and somatostatin receptors and most recently those ligands targeting the misfolded amyloid protein, are good examples of the progress achieved.

**Table 2:** **The early years of nuclear medicine**

1934	First radioactive ^128^I	Enrico Fermi	Italian physicist	1901 to 1954
1936	Production of ^99m^Tc	Emilio G. Segre	Italian physicist	1905 to 1989
1936	First therapy with ^32^P	John H. Lawrence	American physicist	1904 to 1991
1938	Discovery of ^131^I	Glenn Seaborg	American chemist	1912 to 1999
1942	Therapy of benign thyroid disease	Saul Hertz	American physician	1905 to 1950
		Robley D. Evans	American physicist	1907 to 1995
1946	First therapy of thyroid cancer	S. M. Seidlin	American physician	1895 to 1955
		Leo D. Marinelli	American physicist	1886 to 1995
1949	First therapy of thyroid			
	Carcinoma in Europe	Cuno Winkler	German physician	1919 to 2003
		Eric E. Pochin	British physician	1909 to 1990

Whilst on the topic of interdisciplinarity, it is opportune to underline that not only physicists greatly contributed to the development of Nuclear Medicine. This equally applied *inter alia* to engineers, chemists and radiopharmacists. Gopal Subramanian with a degree in chemical engineering introduced ^99m^Tc labelled phosphonates for bone scanning; Hal Anger as an electrical engineer and biophysicist developed the Anger gamma camera; Roger Ekins, also a biophysicist, developed the saturation analysis/radioimmunoassay methodology (Table 3). Physicists turned physiologist developed and emphasised the need for elegance and simplicity in quantitative measurements.

**Table 3:** **Twenty-five years of seminal discoveries**

1951	Rectilinear scanner	Benedict Cassen	American physicist	1902 to 1972
1953	CBF with radio krypton	Niels Lassen	Danish physician	1926 to 1997
1958	Anger gamma camera	Hal O. Anger	American engineer	1920 to 2005
1959	Radioimmunoassay	Rosalin S. Yalow	American physicist	1921 to 2011
		Solomon Berson	Americal physician	1918 to 1972
1960	Saturation analysis	Roger Ekins	British biophysicist	b. 1936
1962	Tc-99 m generator	Paul Harper	American surgeon	1915 to 2005
		Katherine Lathrope	American physicist	1915 to 2005
1962	SPET	David Kuhl	American physician	b. 1929
1971	Polyphosphates	Gopal Subramanian	American chemist	1953 to 2000
1973	PET	Michel Ter-Pogossian	American physicist	1925 to 1996
		Michael Phelps	American biophysicist	b. 1939

From the preceding pgraph, it is clear that fundamental discoveries spanned a period of a century. In the space available for this short piece, it is simply not possible to give due consideration to all those many eminent scientists who developed the field. So I shall focus on the three seminal physics developments which fundamentally changed the practice and future of Nuclear Medicine: the introduction of the rectilinear scanner, the development of the gamma camera and, finally, the design of the first single-photon emission computed tomography (SPET), positron emission photography (PET) and PET/computed tomography (CT) instruments.

Surface counting had been an important milestone in the clinical development of the radioactive tracer method. It was used early on by Norman Veal and others in mapping the placenta, the thyroid and the pericardium. This was laborious, a manual-driven process and rather time-consuming. It was difficult to perceive much more than the simplest outlines of organs, and yet, quantitative measurements were already taking place.

The relationship between physicists and physicians has always been most interesting. A healthy diffidence between both experts was often present and wonderfully illustrated from the following extract, taken from the outstanding chronology authored by Marshall Brucer, the first President of the Society of Nuclear Medicine (USA) and Chairman of the medical division of Oakridge Institute of Nuclear Studies from 1948 to 1962. And one can read in page 291: ‘…three months after the London meeting (the first meeting at University College London, on 29th July 1950, where data from ^131^I therapy was discussed)’, Sam Seidling asked: ‘If a metastasis has high uptake, we can destroy it. Now, for God’s sake, when will physicists learn to measure ^131^I uptake?’. Leo Marinelli murmured: ‘As soon as physicians decide how much uptake is high’.

Benedict Cassen changed all this with his discovery of the rectilinear scanner in 1950. Born in New York in 1902, he graduated in physics and mathematics from the Royal College of Science in London in 1927. He moved to the California institute of Technology in 1930. Imaging the thyroid, he reported first results in 1950. The radioactive tracer method would forever be linked to imaging, for better or for worse. Single-head, dual-head, 3-inch or 5-inch rectilinear scanners and whole-body scanners became routine imaging instruments for 2D organ imaging and counting, dominating for some 30 years Nuclear Medicine applications well into the late 80s, as new tracers became available.

Whilst brilliant individual scientists made major contributions to medicine, institutions and or societies have a habit of getting it wrong. In the late 40 s, much debate and thought went into what role a medical physics department should have in a hospital. To quote an example: ‘…Any one radio-element investigation may be too short lived to justify the provision by the department concerned of the best apptus for the job’. [4]. Whilst Cassen had already proven that this viewpoint was completely wrong, how would Hal Anger comment on the on the previously mentioned discussion?

## US patent 3,011,057 in 1961

Nuclear Medicine was to change forever with the mentioned patent, defining the Anger gamma camera. It is truly astonishing that this technology, still in worldwide use today, has outlived over half a century of technological breakthroughs and progress! In modern times, there is probably no other example of such a long lasting instrumentation breakthrough.

**Figure 2:**
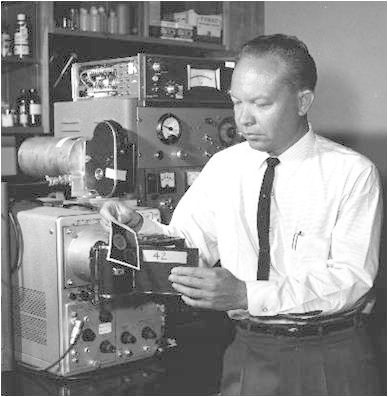
**Hal Oscar Anger (1920 to 2005).**

Hal Oscar Anger (Figure 2) was born on the 20th of May 1920, in Denver, USA. He graduated as an electrical engineer. His innovative career spanned from radar jamming equipment to radiation detector devices, culminating with his seminal invention of the imaging device still in use today. His camera was presented at the 1958 meeting of the Society of Nuclear Medicine and led to an explosion of commercial exploitations. The history of the patent itself would merit a septe chapter. He died on October 21, 2005, in Berkeley. No writings can give sufficient justice to Anger's innovative genius and the impact he has had worldwide on millions of patients investigated with his seminal instrument.

**Figure 3:**
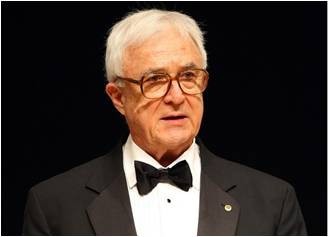
**David Kuhl (born in 1929, retired in 2011).** Image taken during presentation of the 2009 Japan Prize Award, attended by Emperor Akihito. This prestigious prize was introduced in 1985 to award scientists who contribute to the development of science and technology.

It would take some 30 years before 3D Imaging became an integral part of the development of Nuclear Medicine. And one has to give proper due to a medical scientist, David Kuhl (Figure 3), the originator of single-photon emission tomography. Born in St. Louis, Missouri in 1929, David E. Kuhl graduated in medicine at UPENN in 1955. In 1964, David Kuhl and Roy Edwards developed the Mark II emission tomographic scanner, starting the field of cross-sectional tomographic imaging. Kuhl went to develop the technique of SPET - truly ahead of its time and ahead of the development of the CT scanner (1973). Should one write a history of missed Nobel awardees? Mark II was followed by Mark IV and a number of subsequent improvements.

Single-slice SPET imaging was subsequently superseded by whole-volume SPET imaging, with the introduction of the rotating gamma camera (Anger's device, shaping *de novo* the clinical applications of the radioactive tracer method). Without SPET, nuclear cardiology, and less so, neurotransmission imaging would have not risen to the clinical pre-eminence these modalities reached in the last 15 years. SPET became a truly worldwide available technology, supported by a range of useful radiopharmaceuticals.

## Modern technologies (SPET, SPET/CT, PET, PET/CT and PET/MR)

The beginnings and development trends of positron emission tomography are outlined in Table 4. Again, constraints on space prevent a detailed analysis. Suffice to say that for a physician, interested in patient care and management, it took a rather long time before clinical useful applications began to emerge [5–7]. It would take the development and final availability of ^18^F labelled glucose, which made positron emission tomography a clinically useful tool. Between the development of the first PET system in 1973 by Michael Phelps and the approval by the FDA of ^18^F labelled glucose, 24 years would have elapsed (Table 4).

**Table 4:** **The history of PET**

1951	First use of NaI probes for positron detection in brain	William Sweet and Gordon Brownell
1963	First description of radon equations for image reconstruction	Alan M. Cormack
1973	Description of CT scanner	Godfrey N. Hounsfield
	First PET tomograph	Michael E. Phelps
1976	First commercial PET scanner	
1978	First BGO-based scanner	Chris Thompson
1977	^14^C Deoxyglucose	Louis Sokoloff
1978	^18^F Fluorodeoxyglucose	Tatsuo Ido
1986	Present synthesis of FDG	Kurt Hamacher
1984	Commercial cyclotron development	
1997	FDA approves FDG as radiopharmaceutical	
1998	PET/CT prototype	David Townsend and Ron Nutt
1999	Lutetium orthosilicate (LSO)	
1999	Medicare reimburses for staging NSCLC, SPN, colorectal ca, HD and NHD, melanoma, hibernating myocardium and TLE	
2001	PET/CT in UK at INM/UCL	
2002	Health technology assessments (HTA) begin	

**Figure 4:**
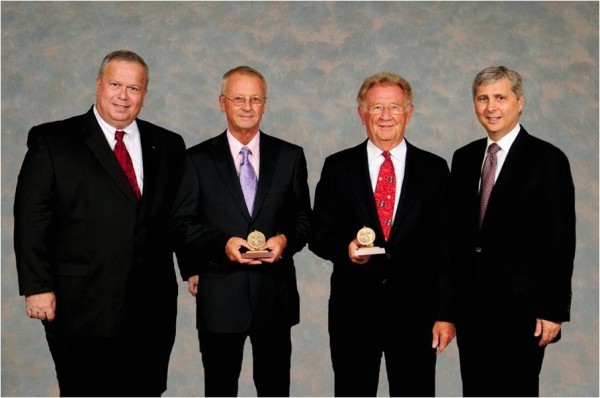
**David W Townsend (2nd left) and Ronald Nutt (2nd right).** The IEEE Medal for Innovations in Healthcare Technology was given to David W Townsend (2nd left) and Ronald Nutt (2nd right). Photograph taken with Moshe Kam (IEEE President Elect, left) and Pedro Ray (IEEE President, right).

To complete this brief review, we end with David Townsend, Ph.D., who gave us the most significant development in medical imaging in the last 10 to 15 years. The PET/CT prototype, attributed to Townsend and Nutt (Figure 4), then President of CPS Innovations, was named by TIME Magazine as the medical invention of the year 2000. A hugely impressive development, bringing human anatomy and biochemistry onto a combined 3D map, this technology was instantly adopted by the medical community as an essential tool for early staging and monitoring of human disease. No hospital facility today can bypass the availability of a PET/CT system for appropriate patient management.

What about PET/MR? Time will tell; its adoption by the medical community will take much longer. But the input of physics computing and engineering will remain vital for the future development of this innovative speciality. This will be ever more relevant as the demands posed by multimodality imaging technologies and the need for true quantitative and reproducible measurements are widely felt. This is especially relevant in the increasing need for the monitoring of interventions, being medical, surgical or pharmacological, applied to an individual patient. Whilst the overtly simplistic aims of personalised medicine are being reassessed, patient specific interventions will grow, and with it, the growth of physics in the field is assured.
